# Arterial dilator function in athletes: present and future perspectives

**DOI:** 10.3389/fphys.2015.00163

**Published:** 2015-05-19

**Authors:** David Montero

**Affiliations:** Zurich Center for Integrative Human Physiology, Institute of Physiology, University of ZurichZurich, Switzerland

**Keywords:** vascular function, conduit artery, resistance artery, blood flow distribution, athletes

## Introduction

Although it is well established that exercise training conditions the vasculature (Joyner and Green, 2009), the status of arterial function in long-term trained subjects (i.e., athletes, ATH) has been recently disputed (Green, 2013; Montero et al., 2013). The controversy arose as to whether vasodilator function was enhanced in ATH compared with untrained healthy peers (Green et al., 2013). This contention was justified by the conflicting report of decreased (Green et al., 2011; Phillips et al., 2011) and normal (Franzoni et al., 2005b; Moe et al., 2005; Naylor et al., 2006; Ballard et al., 2008; Rognmo et al., 2008; Nualnim et al., 2011; Phillips et al., 2011; Rowley et al., 2012; Agrotou et al., 2013; Green et al., 2013) but also increased (Jensen-Urstad et al., 1999; Rywik et al., 1999; Rinder et al., 2000; Franzoni et al., 2005b; Kasikcioglu et al., 2005; Rickenlund et al., 2005; Tanriverdi et al., 2005; Galetta et al., 2006; Hagmar et al., 2006; Walther et al., 2008; Florescu et al., 2010; Nualnim et al., 2011; Welsch et al., 2013) flow-mediated dilation (FMD), a common index of conduit artery dilator function, in ATH vs. age-matched controls. Likewise, the evidence on resistance artery dilator function was apparently heterogeneous (Green et al., 2012). Presumably, differences in methodology, study population and the small sample size of previous reports, among others, may have contributed to cloud the status of arterial function in ATH, leading to wide speculation (Green, 2013; Montero et al., 2013). What follows is a brief viewpoint on the state-of-the-art including comprehensive meta-analytic data spanning through macro- and microvascular dilator function in primarily endurance-trained ATH, as well as an insight in to next challenges to the field.

## Athlete's artery: a superior functional phenotype

Over the inconsistent results of single studies, pooled evidence demonstrates, in a paradoxical manner, an homogeneous enhanced dilator function in the smallest limb arteries and arterioles of ATH, irrespective of the age status (Montero et al., 2014b). This strongly suggests a predominant type II error in prior research, which indeed is likely common to scientific fields based on small sample size studies (Ioannidis, 2005). In addition, it is noteworthy that ATH exhibit enhanced endothelium-dependent and -independent resistance artery dilator function (Montero et al., 2014b), the latter contrasting with the passive role conventionally bestowed on the vascular smooth muscle, albeit concurring with early works (Haskell et al., 1993). Thus, it is uncertain whether the enhanced resistance artery dilator function in ATH is in accordance to improvements in smooth muscle function alone or along with endothelial function, which may not be entirely distinguished *in vivo* (Oltman et al., 1992). Regardless, such enhanced dilator function may correlate with increased basal vascular tone (Sugawara et al., 2007), provided that microvessel density (Lloyd et al., 2003; Zoladz et al., 2005), but not basal peripheral vascular conductance (Di Bello et al., 1996; Higashi et al., 1999; Carrick-Ranson et al., 2014), is increased in ATH.

The superior phenotype of conduit arteries in ATH may be discerned in light of pooled evidence according to age (Montero et al., 2014a). Herein, brachial artery FMD and nitrate-mediated dilation (NMD), respectively reflecting endothelium-dependent and –independent conduit artery dilator function, are enhanced in master (mean age >50 years) but similar in young (mean age <40 years) ATH compared with age-matched controls (Montero et al., 2014a). Conversely, basal brachial artery diameter is increased in young but similar in master ATH vs. age-matched controls (Montero et al., 2014a). Collectively, it can be inferred that both young and master ATH unequivocally demonstrate enhanced peak brachial diameter, since FMD and NMD techniques always characterize the response to a given dilator stimulus relative to basal artery diameter, despite differences may exist concerning the edge detection and wall tracking procedure, cuff-occlusion length/placement and time interval of diameter assessment (Thijssen et al., 2011). Moreover, FMD and NMD may be underestimated in young ATH in view of the widespread inverse correlation between these measures and basal conduit artery diameter (Thijssen et al., 2008). Likewise, the main dilator stimulus triggering FMD, i.e., endothelial shear rate, could be diminished in young ATH due to brachial enlargement (Pyke and Tschakovsky, 2007), which additionally suggests the presence of enhanced brachial endothelium-dependent dilator function in young ATH.

Of note, arterial adaptation in ATH may be regulated in a region-specific manner according to the impact of training characteristics (intensity, duration, frequency, volume, modality) on hemodynamic forces such as shear rate and pressure patterns, nitric oxide (NO) production and vessel structure (Maiorana et al., 2003; Prior et al., 2003a,b). These might fluctuate during periodized training programs and thereby modulate endothelial and smooth muscle dilator function. Furthermore, vasodilation is ultimately a function of the balance among substances and mechanisms that can induce dilation or constriction, including, but not limited to, NO, adenosine, ATP, tissue metabolites, endothelin-1, noradrenaline, inward rectifying K^+^ channels and reactive oxygen species (Korthuis, 2011). In this regard, endothelial dilator function in ATH has been positively associated with plasma antioxidant activity (Franzoni et al., 2004, 2005a), which is subject to diurnal variation (Hammouda et al., 2012). Therefore, the dynamic status of arterial dilator function may have compounded the variability and hence lessened the statistical power of previous studies in ATH. In addition, the majority of preceding research involved lower limb-trained ATH and assessed upper limb arterial function (Montero et al., 2014a,b). Nevertheless, the arterial dilator phenotype of ATH seems to be qualitatively identical but of higher magnitude if determined in primarily trained vs. untrained limbs (Walther et al., 2008). Taken together, the athlete's artery is distinguished by a supra-normal dilator function in resistance and (size-adjusted) conduit vessels, which facilitates conductance and thereby plausibly improves blood flow regulation.

## The unknown optimal dilator-constriction balance

The fact that peripheral vascular conductance during submaximal exercise may not be increased in ATH (Fleg et al., 1994; Carrick-Ranson et al., 2014) suggests that enhanced arterial dilator function interacts with increased arterial constrictor function and/or sympathetic constrictor drive. In this respect, increased both arterial constrictor function and sympathetic (re)activity have been observed in ATH (Furlan et al., 1993; Welsch et al., 2013). Seemingly, the antagonism between dilator and constrictor forces may be futile unless associated with a more efficient perfusion in that part of the cardiac output is diverted from tissue with low oxygen demand toward active skeletal muscle. This would result in increased oxygen extraction and reduced cardiac output/limb blood flow during submaximal exercise, as indeed have been noted at whole body (Ekblom et al., 1968) and limb (Proctor et al., 2001; Lawrenson et al., 2004) levels after endurance training and within exercising muscle in ATH (Kalliokoski et al., 2001).

At maximal exercise, an improved blood flow distribution may, at least in part, explain the increased arteriovenous oxygen difference seen in ATH (Fleg et al., 1994; Mollard et al., 2007a,b; Carrick-Ranson et al., 2014). Importantly, a suboptimal blood flow distribution cannot be compensated at maximal exercise by increasing the cardiac output since the maximal pumping capacity of the heart, and thus systemic oxygen delivery, is reached (Mortensen et al., 2005; Brink-Elfegoun et al., 2007; Calbet et al., 2007; Elliott et al., 2015). Moreover, oxygen extraction is reduced when overall limb vasodilation is experimentally enhanced during submaximal and maximal exercise (Calbet et al., 2006), which supports the necessary role of vasoconstriction in less active tissue to efficiently distribute blood flow. On the other hand, excessive vasoconstrictor activity may increase oxygen extraction in relative terms but concomitantly reduce oxygen consumption (VO_2_) via lower absolute blood flow to active skeletal muscle (Heinonen et al., 2013).

Therefore, the optimal arterial function, as regards exercise performance, must contribute to maximize blood flow to active skeletal muscle and minimize blood flow to other tissues, while maintaining the required perfusion to exercise-related *vital* organs (heart, lungs, skin, brain). This is certainly favored by a region-specific arterial function, as evidenced by the observations that arterial constrictor function is enhanced in the upper limb after cycling training (Rakobowchuk et al., 2012), whereas arterial dilator function is increased in the lower limb but decreased in the upper limb of cyclists vs. swimmers (Walther et al., 2008). In this line, maximal VO_2_ during leg cycle ergometry is more closely associated with arterial dilator function determined in the lower vs. upper limb (Ridout et al., 2005). Accordingly, there must be a combination of equilibria between arterial dilator function and vasoconstriction at distinct (whole body, limb, muscle) levels that will best facilitate exercise performance. However, although major breakthroughs in the knowledge of molecular, biochemical and cellular bases of arterial dilator function during exercise have been witnessed (Pagliaro et al., 1999; Joyner and Wilkins, 2007; Calbet and Joyner, 2010; Hellsten et al., 2012), we are still in the early stages of studies capable of integrating them with concurrent vasoconstrictor activity (Reed et al., 2000; Tschakovsky et al., 2002; Rosenmeier et al., 2004; Mortensen et al., 2012; Heinonen et al., 2013). Undoubtedly, the journey toward understanding the complex way in that arterial function serves athletic capacity has just begun.

## Conclusion

The remarkable arterial dilator capacity demonstrated by ATH is partly attributed to an enhanced response to vasodilator stimuli. This is particularly observed in peripheral resistance vessels, but also in upstream conduit arteries when adjusted for lumen dimensions, which are increased in young ATH. Notwithstanding the age status, the *athletic* arterial phenotype is, at least in part, characterized by the enhancement of vascular smooth muscle dilator function. Yet, such enhanced dilator function seems to be region-specific and paralleled by increased vasoconstriction in order to efficiently distribute a finite cardiac output. The understanding of the interplay between arterial dilator-constrictor function and sympathetic drive at multiple hierarchical levels during exercise remains elusive and a challenge for future research in to the physiological bases of human performance.

### Conflict of interest statement

The author declares that the research was conducted in the absence of any commercial or financial relationships that could be construed as a potential conflict of interest.
